# Intrinsic Functional Connectivity of the Anterior Cingulate Cortex Is Associated with Tolerance to Distress

**DOI:** 10.1523/ENEURO.0277-21.2021

**Published:** 2021-10-13

**Authors:** Or Dezachyo, Stas Kozak, Yair Bar-Haim, Nitzan Censor, Eran Dayan

**Affiliations:** 1 School of Psychological Sciences and Sagol School of Neuroscience, Tel Aviv University, Tel Aviv 6997801, Israel; 2 Department of Radiology and Biomedical Research Imaging Center, University of North Carolina at Chapel Hill, Chapel Hill, NC 27599

**Keywords:** resilience, distress tolerance, functional networks, ACC, functional connectivity

## Abstract

The ability to adapt under significant adversity, defined as psychological resilience, is instrumental in preventing stress-related disorders. An important aspect of resilience is the capacity to endure affective distress when in pursuit of goals, also known as distress tolerance. Evidence that links intrinsic baseline interactions within large-scale functional networks with performance under distress remains missing. We hypothesized that the anterior cingulate cortex (ACC) may engage in distress tolerance because of its involvement in attention and emotion regulation. Accordingly, we tested whether behavioral performance under distress is associated with baseline resting-state ACC functional connectivity (FC). Distress tolerance was measured in 97 participants using the behavioral indicator of resiliency to distress (BIRD) task. Analyses contrasted participants who quit the task before its designated termination (*n* = 51) with those who persisted throughout it (*n* = 46). Seed-based FC analysis indicated greater connectivity between the ACC and dorsolateral prefrontal cortex (DLPFC) in subjects who persisted throughout the task, along with greater FC between the ACC and the precentral gyrus in those who quit before its termination. The results shed light on the mechanisms underlying interindividual differences in the ability to handle distress.

## Significance Statement

The brain mechanisms underlying distress tolerance, the process of withstanding adversity while pursuing a goal, are incompletely understood. Here, we tested whether the anterior cingulate cortex (ACC), a region that integrates sensory input and guides attention to salient stimuli is involved in distress tolerance. Our results reveal increased resting-state functional connectivity (FC) between the ACC and the left dorsolateral prefrontal cortex (DLPFC) in individuals with high level of distress tolerance, and reduced FC between the ACC and the insular and motor cortex in those with lower levels of distress tolerance. Altogether, these results reveal that through functional connections with other cortical regions, the ACC regulates distress tolerance in the healthy brain.

## Introduction

Psychological resilience is widely conceptualized as a dynamic process in which personal or environmental resources are used to adapt in the context of adversity (Kalisch et al., 2015; Bolsinger et al., 2018; Stainton et al., 2019). The study of resilience links psychological, neurologic, and genetic factors with the risk of developing a wide range of psychiatric disorders such as major depression, anxiety, and posttraumatic stress disorder (PTSD; Hjemdal et al., 2011; van der Werff et al., 2013b). A better understanding of psychological resilience and its neural underpinnings can potentially improve treatment development and resilience enhancement programs, and may also inform the selection and training of professionals with elevated risk for traumatic exposure.

One important aspect of resilience is distress tolerance, broadly defined as the capacity to endure affective distress in the pursuit of goals (Trafton and Giffored, 2011). Behavioral persistence when facing distress is derived from the way in which individuals perceive and perform under adversity (Zvolensky et al., 2010), making resilience and distress tolerance conceptually intertwined. A growing body of evidence emphasizes the link between low levels of distress tolerance and the development and perseverance of various psychopathologies (Leyro et al., 2010), such as generalized anxiety (Macatee et al., 2015), depression (Ellis et al., 2013; Allan et al., 2014), and substance abuse (Brown et al., 2002; Daughters et al., 2005; Veilleux, 2019).

Recent research suggests that high levels of distress tolerance mediate resilience by means of cognitive flexibility and emotion regulation (Malooly et al., 2013; Arici-Ozcan, 2019). The ability to endure affective distress requires the suppression of emotionally driven impulses elicited by stressors and is believed to be accomplished via top-downregulation processes (Trafton and Giffored, 2011), whereby cognitive resources are recruited to guide goal-directed behavior exerting effortful cognitive control. Indeed, multiple studies have documented the contribution of cognitive control to distress tolerance (Ledgerwood et al., 2009; Bagge et al., 2013; Macatee et al., 2018). One particular brain region that may mediate distress tolerance is the anterior cingulate cortex (ACC). Constituting a major hub in the salience network (SN), the ACC integrates sensory input and guides attention to salient stimuli (Seeley et al., 2007; Menon and Uddin, 2010), playing a major role in cognitive control and decision-making (Kerns et al., 2004; Kolling et al., 2016; Peters et al., 2016). Work in rodents has also demonstrated a direct role for the ACC in behavioral persistence, showing a decrease in θ power (7–9 Hz) along with an increase in γ power (55–100 Hz) as rats got closer to quitting a task that gradually increased in difficulty (Porter et al., 2020). Moreover, neuroimaging studies point to the involvement of the ACC in PTSD (Sherin and Nemeroff, 2011; Sripada et al., 2012; Gupta et al., 2017), suggesting a role for the ACC in modulating emotional response to stressors (Bolsinger et al., 2018).

**Figure 1.**
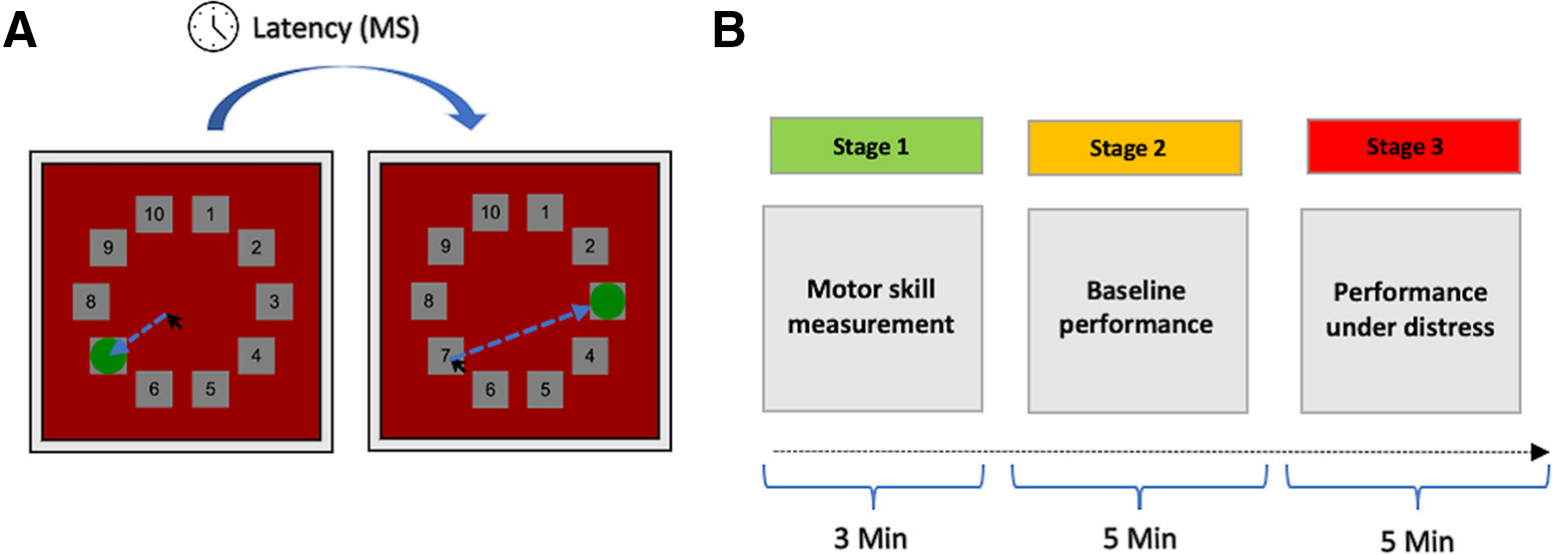
The BIRD task. ***A***, Schematic illustration of a BIRD trial. Participants were presented with a series of numbers arranged in a circle. In each trial, participants were instructed to move the cursor to the number covered by the green dot before the dot moved away and the trial ended. Latency was measured based on trial duration. ***B***, Latency was adjusted to performance at the first stage of the task and was held constant until 1 min before the end of the second stage, when it was reduced by half. Latency at the third stage of the task was the same as that used during the end of stage 2, but participants were given the option to quit at any time.

Distress tolerance varies widely in the general population (Simons and Gaher, 2005; Arici-Ozcan, 2019). Yet, the brain mechanisms that underlie distress tolerance remain largely unknown. Here, we investigated whether intrinsic functional interactions (i.e., functional connectivity; FC) of the ACC mediate distress tolerance in a sample of young healthy volunteers from the Nathan Kline Institute-Rockland Sample (NKI-RS; Nooner et al., 2012). Our analysis focused on participants, aged 18–35 (*N* = 97) who completed the behavioral indicator of resiliency to distress (BIRD) task (Fig. 1; Lejuez CW, Daughters SB, Danielson CW, Ruggiero K, unpublished observations), a common laboratory paradigm that measures distress tolerance. The BIRD task taps into participants’ persistence in a task that gradually increases in difficulty, thus magnifying affective distress. During the last and most distressful phase of the BIRD, participants are allowed to terminate the task before its intended completion, offering an immediate way to reduces affective distress. Participants were classified into two groups, those who persisted in the task until its designated termination (persistent group) and those who quitted the task before its designated termination (quit group). We then delineated differences between the two groups in resting-state FC of the bilateral ACC in comparison to a control region. We expected to find a specific link between distress tolerance and FC of the ACC.

## Materials and Methods

### Participants

Behavioral and imaging data were obtained from the NKI-RS dataset (http://fcon_1000.projects.nitrc.org/indi/enhanced/index.html), a community sample of participants across the lifespan (Nooner et al., 2012). We obtained data from 97 individuals (54 females) aged 18–35 years (mean = 23.75, SD = 4.79), who participated in the study’s discovery phase, completed the BIRD task and had no history of mental illness. All participants provided informed consent, and the study was approved by the local Institutional Review Board.

### Behavioral procedure and analysis

As a measure of distress tolerance, we used subjects’ performance in the BIRD task, collected as part of the NKI-RS protocol. The BIRD is a widely used behavioral task measuring distress tolerance (Daughters et al., 2009; Danielson et al., 2010; Amstadter et al., 2012; Lejuez CW, Daughters SB, Danielson CW, Ruggiero K, unpublished observations) shown to increase self-reported frustration and anxiety as well as physiological arousal, reflected in elevated skin conductance and heart rate (Brown et al., 2002; Leyro et al., 2010). This task is a modification of the paced auditory serial addition task (PASAT), in itself a widely used measure of distress tolerance.

The BIRD task was administered to participants outside of the scanner. Participants were presented with a screen that showed a bird inside a cage and 10 numbered boxes around a circle (Fig. 1*A*). On each trial, a green dot randomly appeared above one of the boxes for a short duration (i.e., latency) before it moved to the next box; participants were instructed to move the cursor and click on the green dot before it moved away from the box. Participants heard a pleasant sound and were awarded with points for each successful trial. If they failed, an unpleasant sound was played (and points were neither awarded nor subtracted). The BIRD is composed of three stages. The first stage (motor skill measurement; Fig. 1*B*) lasts 3 min. It begins with a latency of 5 s but changes in congruence with performance: successful trials reduce the latency by 0.5 s, whereas failure increases the latency by 0.5 s. An average latency is then calculated to assess individual levels of motor skill. In the second stage of the task (baseline performance), which lasts 4 min, participants perform trials with the average latency, as calculated at stage 1. Levels of difficulty increase in this stage, thereby inducing affective distress. Latency is then reduced by half during the last minute of this stage, making the task more distressing. Following a short break, the third stage of the task begins (performance under distress) featuring the diminished latency for a total of 5 min. However, in this stage participants are allowed to quit at any given time by simply pressing a button to end the game, which is displayed on the computer screen.

Tolerance to distress in the BIRD can be measured in two ways (Brown et al., 2002; Amstadter et al., 2012; Daughters et al., 2017). First, it can be defined as a categorical variable, distinguishing between participants who persisted in performing the task during its entire duration (persistent group) and those who quit before the task was terminated (quit group). Second, distress tolerance in the BIRD can be operationalized as a continuous variable, based on the persistence duration (in seconds) at the last stage of the task.

Consistent with the above, group comparisons were based on the categorical variable of persistence, comparing participants in the persistent and quit groups. The two groups were also compared for differences in potential confounding variables, including age, previous exposure to trauma (van der Werff et al., 2013a), and state and trait anxiety (Zvolensky et al., 2010), assessed with the State Trait Anxiety inventory (Kvaal et al., 2005). Group differences in continuous variables were computed using an independent-sample *t* test, while differences in categorical variables were examined using a binomial test. Correlations were computed using the Pearson correlation coefficient. In all tests, significance levels were set at 0.05.

It should be noted that the BIRD task has been typically used with adolescent participants (Daughters et al., 2009; Danielson et al., 2010; Amstadter et al., 2012), whereas its predecessor task, the PASAT, has typically been used in adults. Nevertheless, the proportion of subjects who persisted and quit during the BIRD task (see Results) was similar to that reported in previous studies with adults, where the PASAT was used (Brown et al., 2002; Eichen et al., 2017). This suggests that administering the BIRD was sufficient in inducing the same levels of distress which are characteristic of the PASAT.

### Magnetic resonance imaging (MRI) data acquisition

Imaging data were acquired with a 3T Siemens MAGNETOM Trio Tim scanner. Functional MRI (fMRI) scans were acquired at rest with the following imaging parameters: repetition time (TR) = 1400 ms, echo time (TE) = 30 ms, slice thickness = 2 mm, flip angle = 65°, field of view (FOV) = 224 mm, voxel size = 2.0 × 2.0 × 2.0 mm, slices = 64, acquisition time = 10 min. Anatomical (MPRAGE) T1-weighted images were acquired with the following parameters: TR = 1900, TE = 2.52 ms, slice thickness = 1.0 mm, flip angle = 9°, FOV = 250 mm, voxel size = 1.0 × 1.0 × 1.0 mm.

### Imaging data analysis

Preprocessing of functional images was conducted using the CONN toolbox (version 19.c; Whitfield-Gabrieli and Nieto-Castanon, 2012) and SPM12, both running on MATLAB (version R2020a). Preprocessing included the following steps: realignment, unwarping, slice‐time correction, the segmentation of white matter, gray matter, and CSF, spatial normalization to Montreal Neurologic Institute (MNI) space, and spatial smoothing with a Gaussian kernel of 8 mm full width at half maximum. Denoising was based on nuisance variable regression, wherein signals from the segmented CSF, white matter and six motion parameters (and their first order derivatives) were regressed out of the signal. In addition, outlier volumes (including the single volume preceding the outlier) with excessive head motion were detected with a threshold of 0.9 mm for subject motion and a global signal threshold of *Z *=* *5. Finally, the data were linearly detrended and band‐pass filtered (0.008–0.09 Hz).

### Seed-based FC

To detect group differences in FC of the ACC we subjected the data to seed-to-voxel analysis. The analysis was composed of two levels. In the first level, seed-based correlation maps were computed for each participant based on seed regions of interest (ROIs) in the Harvard–Oxford atlas (Table 1). The maps were computed by correlating the mean time series extracted from each ROI and every voxel in the brain, including cortex, subcortex and the cerebellum. Correlation values were Fisher Z transformed. To test the hypothesis that FC of the ACC mediates distress tolerance, group differences in ACC FC were examined by fitting the data with a random-effect general linear model. Namely, we examined whether the persistent group exhibited greater and/or lesser FC with the ACC than the quit group. The anatomic location of clusters of significant voxels emerging from the group comparisons were identified with the Harvard–Oxford atlas and NeuroSynth (https://www.neurosynth.org/; Yarkoni et al., 2011).

**Table 1:** Seed ROI locations

Coordinates (*x*, *y*, *z*)	Size (mm)	Location	Label
0, +18, +24	5	Anterior cingulate cortex	ACC
0, +43, −18	5	Medial prefrontal cortex	MedFC
+6, +36, +22	5	Right paracingulate gyrus	PaCiG r
−6, +36, +20	5	Left paracingulate gyrus	PaCiG l

To assess whether any of the group differences were specific to the ACC, or rather extended to other brain regions, we conducted a control analysis to test whether the persistent and quit groups differed in FC of the medial prefrontal cortex (mPFC), and the left and right paracingulate gyri, neighboring brain regions which have been widely implicated in various aspects of cognitive control such as decision-making and response conflict (Addicott et al., 2012; Bogerts et al., 2012; Euston et al., 2012; Silva Moreira et al., 2016; Walter et al., 2005). All results were considered significant at a voxel-level threshold of *p* < 0.001, with false-discovery rate (FDR) correction for multiple comparison applied at the cluster-level and set at p-FDR < 0.05.

### Data availability statement

The data that support the findings of this study are openly available in the NKI-RS at http://fcon_1000.projects.nitrc.org/indi/enhanced/.

## Results

### Behavioral results

**Figure 2.**
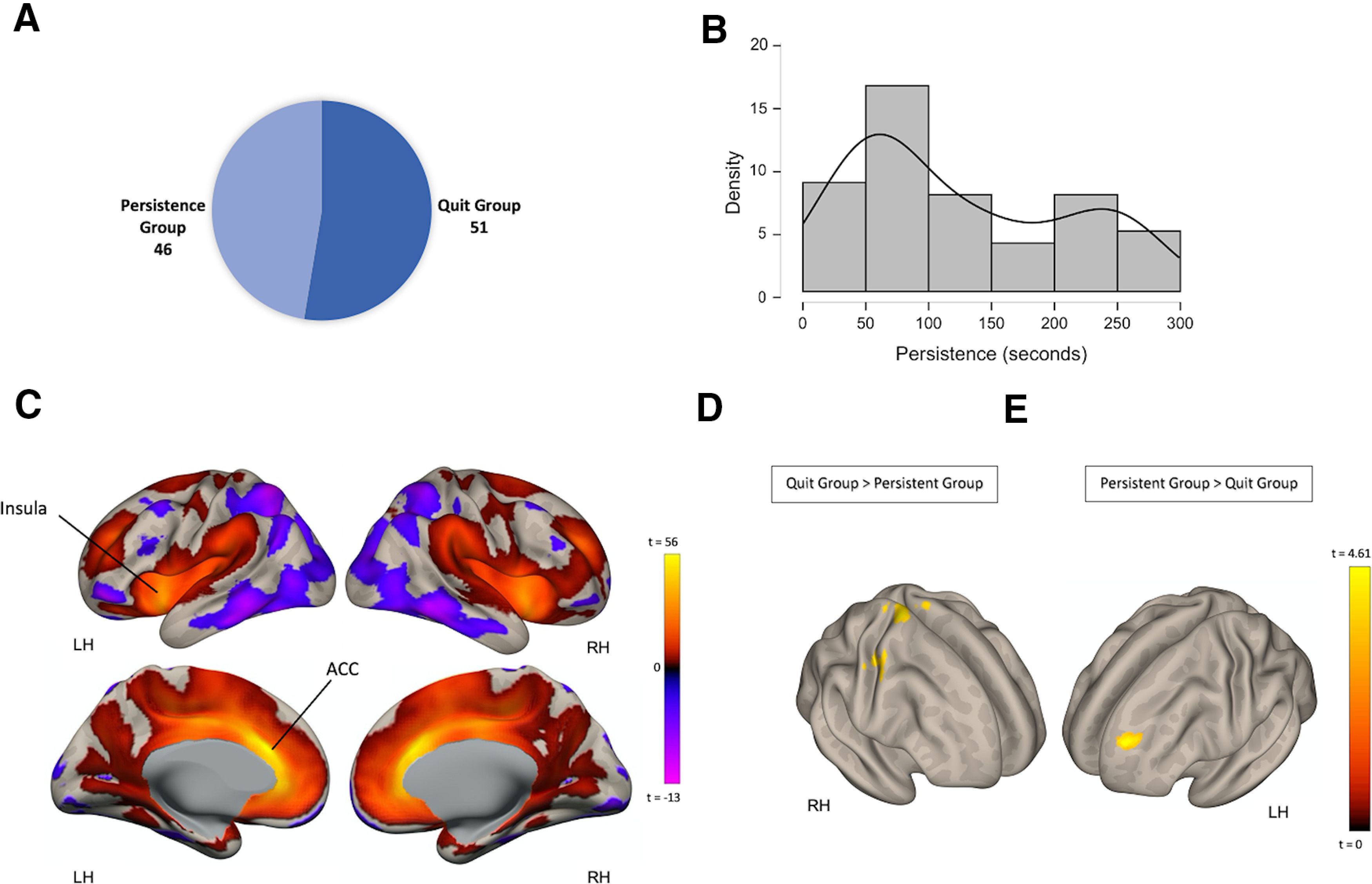
Group differences in ACC FC among subjects who quit and persisted in the BIRD task. ***A***, participants’ classification into two groups: persistent group and quit group. ***B***, Distribution of persistence (quit time) as seen in the quit group. ***C***, Resting state FC between the ACC and the rest of the brain across all participants (*n* = 97). ***D***, Clusters of significant voxels where FC with the ACC was higher in the quit group, relative to the persistent group (quit > persistent). ***E***, Clusters of voxels where FC with the ACC was higher in the persistent group, relative to the quit group (persistent > quit).

Among the sample of 97 participants, 46 (27 females, mean age = 23.83 ± 4.97) have persisted on the BIRD task until its end, while the rest (*n* = 51, 27 females, mean age = 23.68 ± 4.55) quit before termination (Fig. 2*A*). Within the quit group, participants were engaged in the third stage of the task for an average total time of 123.9s ± 84.8, with interindividual differences observed in this variable (Fig. 2*B*). Sex distribution (Z= 0.489, *p* = 0.62) and age (*t*_(95)_ = 0.148, *p* = 0.883) did not differ between the quit and persistent groups. The two groups also did not differ in mean motion during the resting-state fMRI scan (*t*_(95)_ = −0.564, *p* = 0.574), state (*t*_(95)_ = 0.465, *p* = 0.643), and trait anxiety (*t*_(95)_ = 1.062, *p* = 0.291), measured with the State Trait Anxiety Inventory, or in previous traumatic exposure (*t*_(95)_ = 1.062, *p* = 0.897), measured by the total score in the trauma symptom checklist (TSC).

### Analysis of ACC FC

We first tested whether the quit and persistent groups differed in FC of the ACC. To identify the regions which are functionally connected to the ACC, we used a seed-to-voxel approach. Regions showing FC with the ACC across the entire sample of subjects included bilateral cingulate cortex and insular cortex (Fig. 2*C*). Group comparisons were next performed. We first identified clusters where stronger FC with the ACC seed was observed in the quit group, relative to the persistent group (quit > persistent; Fig. 2*D*). This contrast resulted in three clusters spread throughout the cerebral cortex, including in the bilateral primary motor cortex, extending in the left hemisphere to the somatosensory cortex, the left insular cortex and left parietal operculum cortex (Table 2).

**Table 2:** Seed-based FC for the ACC seed

Quit group > Persist group					
Coordinates (*x*, *y*, *z*)	Location	Size	*p* FDR	*p* uncorrected	Cohen’s *d*
+14, −24, +74	Precentral gyrus	406	0.013942	0.000558	0.794
−36, −02, +18	Left insular cortex, left parietal operculum cortex	274	0.039228	0.003138	1.006
+54, −06, +54	Right primary motor cortex, right premotor cortex	244	0.040155	0.004819	0.813
Persist group > Quit group					
−24, +54, +26	DLPFC	228	0.048802	0.006100	0.919

Group differences in ACC FC also existed in the opposite direction. Greater FC in the persistent group, relative to the quit group was found between the ACC seed ROI and a cluster of voxels in the left dorsolateral PFC (DLPFC; Fig. 2*E*; Table 2). These results indicate the involvement of the ACC in distress tolerance. We then investigated whether these patterns of FC were correlated with BIRD task persistence (quit time) in the quit group. Correlations between persistence duration and both ACC-DLPFC FC and ACC-precentral gyrus FC were not significant (*p* > 0.45).

### Seed ROI control analysis

**Figure 3.**
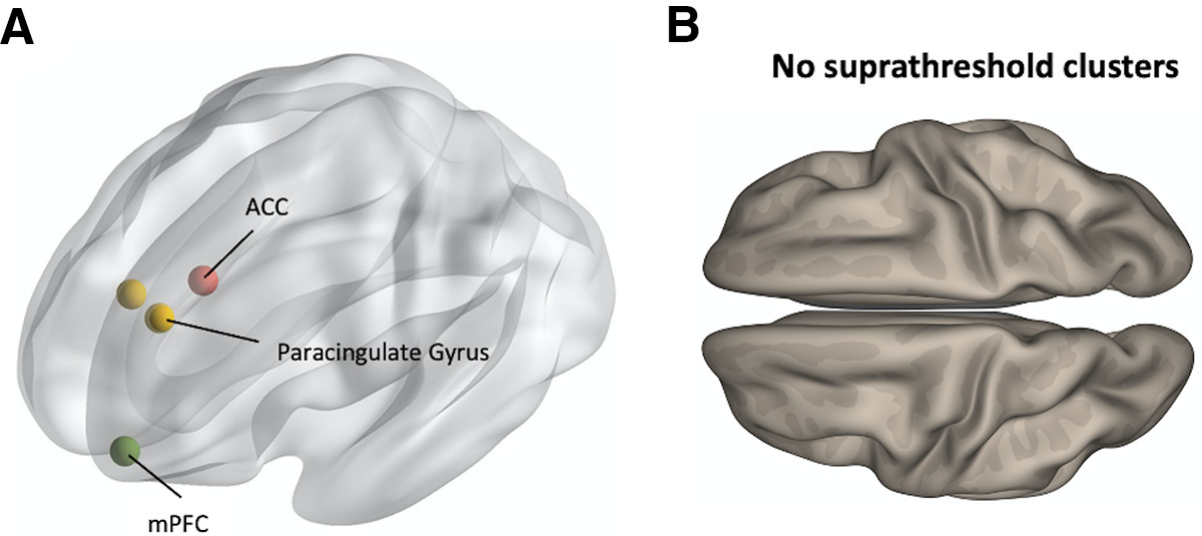
Analysis of control seed ROIs. ***A***, Centroid coordinates of the mPFC and the bilateral paracingulate cortex control seed ROIs. ***B***, Group comparisons in all control seed ROIs yielded no suprathreshold clusters in neither of the contrasts reported in Figure 2 (persistent > quit, or quit > persistent).

To test the specificity of the reported group differences in ACC FC, we repeated the same group comparisons between the quit and persistent groups, with three control seed ROIs: the mPFC and bilateral paracingulate gyri (Fig. 3*A*). These seeds were chosen as spatially-adjacent control ROIs (Dayan et al., 2014; Hoffmann et al., 2016; Table 1), which enabled us to assess the spatial specificity of the findings obtained for the ACC seed. Moreover, the mPFC and the paracingulate gyrus are involved in similar functions, including cognitive flexibility and executive function (Walter et al., 2005; Addicott et al., 2012; Bogerts et al., 2012; Euston et al., 2012; Silva Moreira et al., 2016), which allowed us to examine the functional specificity of the results. Neither of the contrasts reported above (quit > persistent, or quit < persistent) yielded suprathreshold clusters when assessing FC with any of the control seed ROIs (Fig. 3*B*).

## Discussion

Our aim was to examine the involvement of the ACC in distress tolerance. More specifically, we tested the hypothesis that functional interactions involving the ACC are part of the process of withstanding distress in healthy participants who completed a session of resting-state fMRI and the BIRD task, a widely used task measuring distress tolerance. We compared the strength of ACC FC in two groups, those who persisted in the BIRD task and those who quit the task before its designated termination. The quit group exhibited stronger FC between the ACC, and two clusters in the primary motor cortex, as well as greater FC between the ACC and the insular cortex. In contrast, the persistent group showed greater FC between the ACC and the left DLPFC. Differences in FC between the groups were specific to the ACC, and were absent in the mPFC and the paracingulate gyri, neighboring non-overlapping control regions. Furthermore, the quit and persistent groups did not differ in state/trait anxiety, or previous exposure to trauma, further suggesting that the reported differences in ACC FC relate to interindividual differences in distress tolerance, rather than other potential confounding factors.

Our findings indicate involvement of the ACC in distress tolerance, and are consistent with a recent study that used a task-based fMRI design to examine the neural correlates of distress tolerance among substance users and healthy controls (Daughters et al., 2017). This study found that FC among substance users between subgenual ACC and VMPFC was positivity correlated with persistence in a similar distress tolerance task. Moreover, task-elicited activation among substance users was observed in multiple brain regions including the ACC and the right insula. The current findings join these observations, further establishing the involvement of the ACC in distress tolerance in the general population.

The ACC is a key node in the SN, a large-scale brain network comprised primarily of the dorsal ACC and bilateral insula. According to an influential model of the SN, the anterior insula receives input from multiple sensory areas to detect salient environmental events and then recruits the ACC to access higher neocortical areas to ultimately guide behavior (Rudebeck et al., 2008; Menon and Uddin, 2010). Our data point to a link between distress tolerance and FC, both between the ACC and the insular cortex, and between the ACC and the motor cortex. This finding may reflect differential levels of connectivity, both within the key nodes of the SN and between the SN and sensorimotor networks in individuals with low distress tolerance. Of note, the location for the ACC seed ROI used in the current study was anatomically informed. Thus, any inferences regarding large-scale network connectivity and functionality should be made with caution. Nevertheless, we also note that the ACC seed used in our study is spatially adjacent to SN parcels in functionally informed parcellations (Seitzman et al., 2020).

We found that FC between the ACC and the left DLPFC was higher in the persistent group, relative to the quit group. The ACC has reciprocal connections with the frontal lobe which plays a major role in executive functions, response inhibition, and cognitive control (MacDonald et al., 2000; Stevens et al., 2011). Our finding of greater connectivity between the ACC and left DLPFC among subjects in the persistent group suggest the contribution of cortico-limbic inhibition to distress tolerance. This is in line with a recent study that linked cognitive control to distress tolerance (Macatee et al., 2018).

Recent work has linked distress tolerance to psychological resilience (Arici-Ozcan, 2019). The psychological and neurobiological factors that allow certain individuals to function properly under adversity is a topic of significant recent interest (Kalisch et al., 2017; Bolsinger et al., 2018). Studies on resilience have primarily focused on differences between clinical and non-clinical samples (New et al., 2009; Ben-Zion et al., 2020). As a result, our knowledge on the neurobiological substrates underlying resilience in the absence of traumatic events or a neuropsychiatric pathology is limited (van der Werff et al., 2013b; Kalisch et al., 2015; Bolsinger et al., 2018). A recent study found a positive association between resilience among healthy non-traumatized participants, as measured using self-report scales, and gray matter thickness in the right ACC (Gupta et al., 2017). These results are in congruence with the finding reported here, highlighting the importance of functional interactions involving the ACC in distress tolerance in non-traumatized individuals.

Several limitations should be noted when considering the current findings. First, the study is based on a non-clinical sample, thereby limiting our findings and interpretations to healthy populations. Furthermore, our findings are cross-sectional, and cannot be used for making inferences about the role of the ACC in resilience against stressors, and on the propensity of individual subjects to develop psychopathology. It should also be noted that the current study is based on a brain-behavior task-free design. By using a task-free, rather than a task-based design we minimized cognitive factors that might confound distress tolerance and affect task-elicited brain activation such as motivation or working memory. However, we acknowledge that a task-based design could complement the approach used here, and this should be considered in future research.

Taken together, our findings documented a link between distress tolerance and FC of the ACC. These findings reflect the contribution of intrinsic baseline interactions in the ACC to variability in the propensity of healthy non-traumatized individuals to handle distress.

## Synthesis

Reviewing Editor: Ifat Levy, Yale University School of Medicine Decisions are customarily a result of the Reviewing Editor and the peer reviewers coming together and discussing their recommendations until a consensus is reached. When revisions are invited, a fact-based synthesis statement explaining their decision and outlining what is needed to prepare a revision will be listed below. The following reviewer(s) agreed to reveal their identity: Silvina Horovitz, Teddy Akiki.

In this paper, the authors used data from the NKI-RS dataset to study how the outcome of the behavioral indicator of resiliency to distress (BIRD) task relates to resting state networks. They used premature termination in the final phase of the task as a surrogate for tolerance to distress, to categorize participants into two groups (Persistent group: n = 46; Quit group: n = 51). The authors then compared the resting-state functional connectivity of the bilateral ACC between the two groups.

This is an interesting and timely paper, well written and well illustrated. The methods are sound and are clearly explained and the sample size is large. Fig 1. provides a good visual summary for those unfamiliar with the task. The novel analysis uses a subsample from a publicly available dataset, and the results are informative, as they identify a putative marker for tolerance to distress. Additional analyses, however, are needed, to ensure the robustness of the results.

- The Harvard-Oxford atlas is not “functionally” informed (https://doi.org/10.1016/j.neuroimage.2017.02.018). That is, the parcels (including the ACC) are not functionally coherent-in fact, the ACC parcel is split into different ICNs (salience and central executive) https://doi.org/10.1038/s41598-019-55738-y.

The authors should repeat the analyses using an atlas that incorporates fMRI data, whose parcels represent more distinct functional entities. For example: https://doi.org/10.1038/nature18933

https://doi.org/10.1093/cercor/bhx179

- Please add coordinates/size for the seed ROIs. Were the ROIs defined bilaterally?

- Are there measures of anxiety and autonomic system response available for these subjects?

- BIRD modified version of PASAT: Was the persistence duration evaluated for the Quit group? Was there a significant correlation between the persistence time for the quit group and the connectivity between the ACC and the reported areas (motor cortex, DLPFC)? Some subjects almost completed the task while most quit quite early. Figure 2 show there is a distribution of the data which will allow for further exploration.

- The mPFC analysis is helpful, but it would be informative to see results from another 1-2 adjacent parcels as well, to make sure this result wasn’t idiosyncratic.

- It seems that the analysis was limited to the cerebral cortex; subcortex and cerebellum are excluded. If so, this should be noted as a limitation. Most mentions of “brain” ought to be replaced by cortex (e.g., [...] each ROI and every voxel in the brain).

- The fact that both groups are matched with regard to age, sex, and in-scanner motion is reassuring. Are there other relevant variables in the dataset that can be used to explore potential confounds between the Persistent and Other groups? If so, perhaps this could be elaborated in the supplementary table.

Minor comments

- Mention the ROI labels of the Harvard-Oxford parcels that you used. Was “Cingulate Gyrus, anterior division” used for ACC? What about the mPFC?

- Please indicate if the results from the BIRD task were already reported? Add a reference

- Acquisition parameters: TR is reported to be 1400ms (add units) and 64 slices collected - was multi-band used?

- Figure 2: B: add scale to the density.
